# A Systematic Review to Reassess the Formula for Early Life Growth

**DOI:** 10.1016/j.advnut.2026.100660

**Published:** 2026-05-22

**Authors:** Kanishka N Nilaweera, Douwe van Sinderen, Paul D Cotter

**Affiliations:** 1 Department of Food Bioscience, Teagasc Food Research Centre, Moorepark, Cork, Ireland; 2 VistaMilk Research Centre, Teagasc Food Research Centre, Moorepark, Cork, Ireland; 3 School of Microbiology, University College Cork, Cork, Ireland; 4 APC Microbiome Ireland, University College Cork, Cork, Ireland

**Keywords:** breastfeeding, suckling, lactation, energy balance, α-lactalbumin, glucagon-like peptide

## Abstract

Mammals have species-specific adaptations to sustain growth during the suckling stage of life. These include species-specific milk composition and associated whey protein (WP)-to-casein (CN) ratio. Consuming milk (proteins) from other species may therefore cause different growth outcomes during early life. This systematic review examined the effect of intake of feeds with a high bovine WP-to-CN ratio on growth outcomes in infants, piglets, and rodent pups, focusing on milk and/or protein intake, body weight, and gut morphology and its cellular activity. The relevant studies were identified from PUBMed^R^ using the keywords, “whey,” “infant formula,” and “growth” for infant studies and “whey” and “suckling stage” for animal studies. Of the original research articles reviewed (280 in total), 16 infant (with 3207 participants) and 13 animal studies met the imposed inclusion criteria and were independently reviewed by the authors. Data show that consumption of infant formula with bovine whey-to-casein ratio equal to or exceeding that of human milk (60:40), and with proteins supplying ≥8% of total energy, is associated with a discordance between energy intake and weight gain, consistent with net nutrient loss. Data from porcine and rodent models further indicate that the nutrient loss occurs because of reduced nutrient absorption through the gut, and that the effect appears to be driven primarily by bovine WP. We present further evidence supporting a role for gut microbiota in mediating the reduced nutrient absorption and that hydrolysis of bovine WP mitigates, at least some of, this effect and increases growth compared with formulas with intact proteins. We envisage that these findings, based on proteins sourced from cow’s milk, will inform improvements in infant formula that increases nutrient absorption and support healthier growth using a protein content much lower than the current regulatory threshold of 1.8 g/100 kcal.

**Statement of Significance**

Ingestion of infant formula enriched with bovine whey proteins leads to reduced nutrient absorption. Inclusion of hydrolyzed whey proteins in the formula mitigates, at least some of, this reduced absorption and increases body weight gain compared with consumption of intact proteins.

## Introduction

Each mammalian species has developed mechanisms to sustain early life growth when the young are most vulnerable to predation and/or diseases. These mechanisms include species-specific milk composition and specific digestive characteristics of the host. In addition, mammalian species differ in gut microbiota composition, which together with intrinsic factors (e.g., digestive enzyme activities) enable species-specific digestion and absorption of nutrients in milk, ultimately influencing growth rates (TABLE 1, TABLE 2) [[1], [2], [3], [4], [5], [6], [7]]. Thus, it is reasonable to assume that cross-species transfer of milk components should have different outcomes for recipient species compared with ingestion of their own (species-specific) milk and associated nutritional components. This view is supported by the finding that intake of infant formula (IF) created using nonhuman milk components causes increased weight gain in infants when compared with those that are breastfed [[8], [9], [10]]. Although this growth-enhancing effect has been associated with high protein content in IF and the protein effects on growth axis and/or insulin production [[11], [12], [13], [14], [15], [16]], there is still limited understanding of the impact of individual milk components of nonhuman origin on growth outcomes of infants. Our focus here was to fill a knowledge gap of how different ratios of bovine whey proteins (WPs) and casein (CN) used in IF, including those matching human milk (60:40), affect growth outcomes of infants. Our interest to explore effects of different ratios of milk proteins stems from an observational study, which investigated the feeding practices of 1291 infants. The study found that “the most common reason reported by parent(s) for changing their infant’s formula to a non-whey-based formula was that they perceived their baby as being hungry” [17].) Notably, of the 62.4% of parents who gave their 2-mo-old infants whey-based formula, ∼34% reported signs of hunger; consequently, the mothers changed the formula to a nonwhey-based formula (e.g., soy-based) [17]. This observation could be due to an inadequate intake, perhaps due to taste-issues of formulas [18] and/or reduced nutrient (energy) absorption in infants fed high WP-based IF compared with low WP protein IF, who still achieve a growth trajectory much higher than infants offered human milk [8,16]. In support of the latter suggestion that bovine WPs cause a reduced nutrient absorption, we showed that adult mice fed a bovine WP-enriched solid diet have reduced gut weight and length and reduced ileal expression of fatty acid transporter 4 and neutral amino acid transporter, SLC6a19, as well as increased fecal content of triacylglycerols and reduced weight gain compared mice fed CN [[19], [20], [21]]. These changes were accompanied by altered composition and functional potential of the gut microbiota and altered caecal abundance metabolites [19], which we suspect reduced the gut capacity to absorbed nutrients and in turn, also presumably reduced gut weight in mice fed bovine WPs compared with controls fed CN. To obtain further evidence that consumption of feeds containing bovine WPs impact on growth-related parameters, our review of data extended to include other mammals, specifically pigs, mice, and rats during their suckling stage of life and as ways to elucidate the potential mechanisms of action. The findings of this article are particularly relevant to humans as current IFs primarily use bovine milk proteins as key ingredients despite limited understanding of the cross-species impacts of proteins on growth outcomes of infants.

**TABLE 1:** Similarities and differences in early life growth and nutrition

	Bovine	Human	Porcine	Rat	Mouse
Genome size (Gb)	2.8	3.1	2.5	2.8	2.7
Birth weight (g)	30,000–40,000	2500–4500	1000–1800	5.1–6.4	1–2
Growth-rate during suckling period (g/d)	600–800	10–50	200–300	1.3–2.6	0.3–0.4
Growth-rate (g/d) from birth (g/g of body weight at birth/d)	0.015–0.02	0.002–0.011	0.11–0.16	0.2–0.4	0.15–0.2
Milk proteins (g/L)	30–39	7–16	50–60	69–118	34–120
WP (g/L)	5–7	6–8	28–45	9.2–25	13–46
CN (g/L)	24–28	2–4	5–31	64–80	20–73
WP:CN	20:80	Early (90:10 –71:29), mid (60:40) to late (50:50)	90:10 to 47:53	30:70–40:60	39:61
Major WP (% total milk proteins)					
β-Lg	10%	Absent	25%	Absent	Absent
α-LA	3.5%	40%	6%	Present^1^	Present^1^
Predominant Ig	IgG and IgA	IgA and IgM	IgG and IgA	IgG and IgA	IgA and IgG
Minor WPs					
LF	0.5%	30%	0.5%	Present^1^	Present^1^
SA	1%	6%	13%	Present^1^	Present^1^
CNs (% total milk proteins)					
α_S1_CN	33%	11%	Present^1^	Present^1^	Present^1^
α_S2_CN	9%	Absent	Present^1^	Present^1^	Present^1^
β-CN	27%	56%	Present^1^	Present^1^	Present^1^
κ-CN	10%	2%	Present^1^	Present^1^	Present^1^
% amino acid homology to bovine					
β-Lg	100	0	62	0	0
α-LA	100	74	73	63	60
LF	100	69	72	58	63
SA	100	76	80	70	69
α_S1_CN	100	27	41	20	27
α_S2_CN	100	0	62	22	23
β-CN	100	50	61	33	32
κ-CN	100	51	50	35	34

The amino acid sequences correspond to NCBI accession numbers: X14712 (bovine *β*-Lg), X54976 (porcine *β*-Lg), J05147 (bovine α-LA), J00270 (human α-LA), NM_214360 (porcine α-LA), NM_012594 (rat α-LA), NM_010679 (mouse α-LA), M63502 (bovine LF), M83202 (human LF), M81327 (porcine LF), NM_001106864 (rat LF), D88510 (mouse LF), Y17769 (Bovine SA), AF542069 (human SA), NM_001005208 (porcine SA), NM_134326 (rat SA), AJ011413 (mouse SA), M38641 (bovine α_S1_CN), X98084 (human α_S1_CN), X54973 (porcine α_S1_CN), NM_138874 (rat α_S1_CN), M36780 (mouse α_S1_CN), M16644 (bovine α_S2_CN), X54975 (porcine α_S2_CN), NM_001105741 (rat α_S2_CN), D10215 (mouse α_S2_CN), M15132 (bovine *β-*CN), X17070 (human *β*-CN), X54974 (porcine *β*-CN), J00711 (rat *β*-CN), X04490 (mouse *β*-CN), M36641 (bovine κ-CN), M73628 (human κ-CN), X51977 (porcine κ-CN), K02598 (rat κ-CN) and M10114 (mouse κ-CN). Amino acid sequences were compared using CLUSTALW Multiple Sequence Alignment software.

Abbreviations: α-LA, α-lactalbumin; *β*-LG, *β*-lactoglobulin; CN, casein; Ig, immunoglobulin; LF, lactoferrin; NCBI, National Center for Biotechnology Information; SA, serum albumin; WP, whey protein.

1 Data not available.

**TABLE 2:** Digestive characteristics of suckling young

Digestive characteristics	Bovine	Human	Porcine	Rat	Mouse
Morphology					
Total intestine length	23 m (1 M)	3.85 m (birth)	2.8 m (birth)	0.39 m (7 d)	0.19 m (2 W)
Gut transit time	16–30 h	10–30 h (10 M)	24–30 h	2.5–5 h (10 d)	?
Stomach	Subdivided	Glandular (+)	Glandular (+)	Glandular (±)	Glandular (±)
Caecum	Large	Small	Large	Large	Large
Colon	Divisions	Divisions	Divisions	Smooth	Smooth
Appendix	No	Yes	No	No	No
Primary location of fermentation	Rumen	Colon	Colon	Caecum	Caecum
Protein digestion					
Pepsin	+	+	+	+	+
Trypsin	+	+	+	+	+
Carboxypeptidase	+	+	+	+	+
Aminopeptidase	+	+	+	+	+
Chymosin	+	−	+	+	+
% of species-specific protein remaining in raw milk after digestion with adult human gastric and duodenal juice^1^					
*β*-Lg	64%	—	Not available	Not available	Not available
α-LA	91%	92%			
LF	6%	0%			
SA	26%	34%			
CN	4%	5%			
Gut microbiota composition					
Abundant phyla	Bacillota (31%–80%) Actinobacteria (10%–32%) Bacteroidota (9%–25%) Pseudomonadota (7%–17%)	Actinobacteria (45%–60%) Bacillota (20%–40%) Pseudomonadota (7%–12%) Bacteroidota (5%–10%)	Bacillota (40%–80%) Pseudomonadota (8%–12%) Bacteroidota (<5%) Actinobacteria (<5%) Spirochetes (<5%)	Bacteroidota (60%) Bacillota (30%) Verrucomicrobia (<10%) Pseudomonadota (<1%)	Bacteroidota (50%) Bacillota (48%) Actinobacteria (0.4%) Pseudomonadota (0.2%)
Abundant genera	*Lactobacillus,Bifidobacterium, Bacteroides,* *Escherichia_Shigella*	*Bifidobacterium,* *Clostridium,* *Escherichia-shigella, Klebsiella*	*Lactobacillus* *Bacteroides*, *Escherichia-Shigella*, *Lachnoclostridium*	*Bacteroides,Lactobacillus*, *Blautia, Klebsiella*	*Bacteroides*. *Lactobacillus*, *Coriobacteriaceae*, *Shigella*

Abbreviations: α-LA, α-lactalbumin; *β*-LG, *β*-lactoglobulin; CN, casein; LF, lactoferrin; SA, serum albumin; WP, whey protein.

1 Data from [7].

## Milk Composition in Humans and Other Species

Human milk has a relatively low protein concentration when compared with milk of most other mammalian species (Table 1) [5,[22], [23], [24], [25], [26], [27]]. In human milk, protein levels vary during lactation, being highest at early lactation (14-16 g/L) and then decrease as lactation progresses (7–8 g/L beyond 6 mo) [[28], [29], [30], [31]]. The milk protein pattern during lactation mirrors protein requirements of infants ranging from 2.5 g/kg/d in the first month to 1.3 g/kg/d after 4 mo after birth [[28], [29], [30], [31]]. Human milk has a relatively high WP-to-CN ratio, being highest at early lactation (90:10), then progressively declining to 60:40 by mid-lactation and even further at late lactation [29,32], which is similar to porcine milk [33,34]. In contrast, the CN content in milk of other mammalian species such as cows, rats, and mice are higher than the WP content (Table 1) [23,25,27,35]. Other differences include absence of particular WPs (e.g., absence of *β*-lactoglobulin in human and rodent milk [25]), differences in abundance of individual proteins [25,[34], [35], [36], [37]] as well as amino acid sequence-differences of individual proteins [38] (Table 1).

## Digestive Characteristics of Humans and Other Species

There are various similarities yet also differences in gut morphology, digestive enzyme activity, and food transit time between mammalian species (Table 2) [[39], [40], [41], [42], [43], [44], [45], [46], [47], [48], [49], [50]]. When combined with species-specific milk protein composition (Table 1), ingestion of proteins of another species is expected to produce distinct digestive profiles. This suggestion is further supported by the finding that digestion of bovine milk proteins by human digestive enzymes creates distinct protein profiles in the digesta compared with digestion of own (human) milk proteins (Table 2) [7]. Differences also exist in the composition of the gut microbiota between the young of various mammalian species, presumably due to differences in the gut environment and milk composition. For instance, in calves, members of the phylum Pseudomonadota are present in high relative abundance immediately after birth and, over time, as milk intake progresses, members of the phyla Bacillota and Bacteroidota start to dominate (Table 2) [[51], [52], [53]]. Similar to calves, in most other species of mammals, Bacillota dominate the gut environment with compositional fluctuations during the suckling stage [[54], [55], [56], [57]]. Although the infant gut is colonized early by Pseudomonadota, Actinobacteria, and Bacillota [[58], [59], [60], [61]], their composition varies by other factors beyond milk composition including mode of delivery, gestation age at birth, maternal diet, and environment [58]. With regard to infant nutrition, breastfed infants are colonized by members of the genus *Bifidobacterium*. Other genera present include *Lactobacillus* and *Bacteroides*, each varying in abundance [[59], [60], [61]]. In contrast, formula-fed infants have a greater gut microbiota diversity with species belonging to the genera *Staphylococcus*, *Streptococcus*, and *Clostridium* being detected compared with breastfed infants [58,[62], [63], [64]]. Consequently, the fecal metabolite profiles between breastfed and IF-fed infants show differences with Kyoto Encyclopedia of Genes and Genomes (KEGG) pathway, particularly the enrichment of pathways related to fatty acid biosynthesis in infants fed IF [64]. On the basis of the above findings and given the association between infant gut microbiota composition and growth rates [65], it is reasonable to assume that consumption of IF, using proteins originating from cow’s milk, may cause shifts in the gut microbiota composition, with effects that can potentially influence growth outcomes compared with ingestion of own species-specific milk.

## Infant Formula

The parent’s milk represents the best nutritional choice for her nursling [8,66]. In human milk, the protein-associated amino acids and free amino acids are crucial for growth as well as for developing immunity in the young [66,67]. However, breastfeeding is not always possible for a variety of reasons. Thus, the practice of feeding milk of nonhuman origin began, dating back as ≥2000 BC [68]. At present, the majority of IFs on the market use proteins derived from bovine milk as their primary protein source. The preference for bovine milk proteins arises from the high milk volume produced by dairy cows and the greater digestibility of bovine milk proteins compared with other protein sources such as plant proteins [69]. Compared with human milk proteins, however, the bovine milk proteins are digested less efficiently, particularly during the gastric phase, albeit, by the ileal phase, digestion profiles become similar as shown in vitro using the internationally recognized INFOGEST (Improving health properties of FOod by sharing our knowledge on the diGESTive process) digestion models [70]. With regard to the amino acid composition, cysteine and tryptophan are limiting in bovine milk proteins when compared with human milk proteins [16,24,71]. In addition, bovine milk (and some IF) contains fewer free amino acids than human milk [72,73]. Because of the lower digestibility of bovine milk proteins compared with human milk proteins [70] as well as differences in free and protein-bound amino acids in the milk of the 2 mammalian species [16,24,71], a greater amount of bovine milk protein is required in IF to provide the same level of amino acids as human milk (protein) consumption [74]. Thus, a number of regulatory bodies across the world including the European Commission have specified a minimum (1.8 g/100 kcal) bovine protein content with a total energy content between 60 and 70 kcal/100 mL that should be used in the formulation [11,24]. The formulas are adjusted with a bovine WP-to-CN ratio matching that of human milk (60:40) to deliver bovine WP-enriched essential amino acids, which are more readily digested and absorbed into circulation than CN digestion as shown by in vitro studies and after acute in vivo challenges of dietary proteins [[75], [76], [77]]. The allergenicity of the bovine proteins is another consideration in IF manufacture because of their nonhuman origin and the differences in protein composition between bovine and human milk proteins [16,24,71], which are thought to generate adverse immune responses in (some) infants fed IF containing intact bovine proteins [78]. To cater for such susceptible infants, partially hydrolyzed proteins as well as amino acids equivalent to protein content have been developed as hypoallergenic formulations [79,80].

## Impact of Protein Quantity in Breast Milk Compared With IF on Growth-Related Parameters

Comparative analyses of growth trajectory data indicate that infants born in the mid-to-late 20th century exhibited greater weight gain than those born in the late 19th and early 20th centuries [81]. Although there are many reasons for the difference in growth trajectories over these time frames, it is notable that the production and efficient marketing practices of IF show substantial increase in the 20th and 21st centuries, which coincide with a drop-in breastfeeding from 90% in the 20th century to 42% in the 21st century, driven, in part, by the socioeconomic position of the mothers [68,82]. In line with these findings, there is a growing body of evidence suggesting that breastfed infants gain less weight and have a lower risk of development of metabolic diseases such as obesity and diabetes compared with formula-fed infants [12]. Increasing the duration of breastfeeding from <4 mo to >4 mo was associated with a lower BMI at 6 y of age among children who were breastfed for longer [83]. The difference in growth trajectory between breastfed and formula-fed infants has been associated with the difference in circulatory levels of insulin and insulin-like growth factor-1, which are found to be reduced in breastfed compared with formula-fed infants [12,13,16,84]. The hormonal changes are believed to contribute to the altered adiposity (e.g., increased preperitoneal but not subcutaneous adipose) in 5-y-old children who had a history of formula intake, and this adiposity pattern has been associated with increased risk of metabolic diseases later in life compared with children that consumed parent’s milk in early life [11,13,[85], [86], [87]]. Because the minimum protein content of 1.8 g/100 kcal used in IFs, providing ∼7% of energy from proteins has been associated with a higher weight-for-age z (WAZ) score compared with breastfeeding [8,16], and because increasing the protein content in formula from 1.77 to 2.2 g/100 kcal also increased WAZ score of infants consuming the higher protein content [9], it has been recommended that the protein content in IF should be further reduced (e.g., to 1.7 g/100 kcal) [88].

## Impact of Bovine WP-to-CN Ratio on Growth-Related Parameters

Prompted by an observational study reporting signs of hunger in 34% of infants in 62.4% study population receiving bovine WP-enriched formula [17] and by our mouse experiments showing that bovine WPs reduces gut size, ileal nutrient transporter expression, and weight gain compared with bovine CN [19], we hypothesized that consuming feeds enriched with bovine WPs would lead to reduced nutrient absorption. To gather the related data, for human studies, we focused attention on the first year of life. The relevant studies were searched in PUBMed^R^ using keywords (“whey,” “infant formula,” and “growth”), specifically focusing on original research articles that provide information on intake (volume, energy, and/or protein), body weight, and WAZ scores of infants. The studies had to be published and accessible in full text. Studies that had investigated the effects of fixed or different bovine WP-to-CN ratios and in different protein quantities were included in our analyses as ways to assess how each protein combination affects body weight gain. We also reviewed data on the effects of hydrolyzed compared with nonhydrolyzed bovine milk proteins and how inclusion or exclusion of specific proteins in the whey fraction affects growth outcomes of infants. With these inclusion criteria established, the authors independently evaluated 185 research articles, first by screening the title and abstract and then reading the full text to identify potentially relevant articles and those with unclear relevance. The inclusion of studies in the final analysis was jointly established by the authors following resolution of any disagreement. The relevant data were extracted and their accuracy checked independently. Of the 185 research articles reviewed, 16 studies matched the imposed criteria and related data of these studies are shown in Table 3 [[89], [90], [91], [92], [93], [94], [95], [96], [97], [98], [99], [100], [101], [102], [103], [104]]. For animal studies, the focus was on pigs and rodents (mice and rats) during their suckling period. A search was undertaken in PUBMed^R^ using keywords (“whey proteins” and “suckling stage”). In addition to focusing on feed (energy) intake and/or body weight, we also searched for evidence of an impact on the gut as a way to understand the mechanisms for the potential reduced nutrient absorption, which was based on data collected from our rodent studies [[19], [20], [21]]. Similar to the search undertaken for infant studies, the effects of bovine WP-to-CAS ratio and individual WPs in hydrolyzed compared with unhydrolyzed forms on growth outcomes were assessed. The search identified 95 original articles. Review of data allowed us to narrow the list to 13 studies. The related animal studies are detailed in Table 4 [[105], [106], [107], [108], [109], [110], [111], [112], [113], [114], [115], [116]].

**TABLE 3:** Impact of bovine whey proteins-to-casein ratio on growth outcomes of infants

#	Study type	Bovine WP:CN in IF, follow-on milk (FO) or breastfed (BF)	% energy density of proteins	Total energy density (kcal/100 mL)	Age (M)	Outcomes (mo)	Reference
1	Double-blind RCT	IF1: 60:40 (*n =* 82) contain α-LA and PUFA IF2: 60:40 (*n =* 82) contain standard whey BF: 60:40 (*n =* 92)	IF1: 7.6 IF2: 8.8	IF1: 67 IF2: 67 BF:67	1–4	IF2 >IF1 for energy intake (3–4 mo) and protein intake (1–4 mo) but IF2 <IF1 weight gain.	[89]
2	Double-blind RCT	IF1: 71:29 (*n =* 73) added milk fat globular membrane IF2: 62:38 (*n =* 68) BF: (*n =* 72)	IF1: 8 IF2: 7.7	IF1: 60 IF2: 66	2–6	Intake of IF1 >IF2 from 2 to 5 mo but similar WAZ. IF >BF for WAZ.	[90]
3	Double-blind RCT	IF1: 70:30 (*n =* 70) IF2: 70:30 (*n =* 73) BF: (*n =* 65)	IF1: 7.2 IF2: 10.8	IF1: 68 IF2: 68	0.5–12	IF2 >IF1 for energy intake (2–6 mo) and protein intake (0.5–6 mo) whereas body weight increased between 4 and 6 mo and unchanged WAZ up to 6 mo. F2 >BF for weight from 2 to 6 mo.	[91]
4	Blind-2 center parallel	IF1:60:40 (*n =* 29) (industry standard whey) IF2: 70:30 (*n =* 29) (modified sweet whey) IF3: 70:30 (*n =* 27) (acid whey) BF: (*n =* 28)	IF1:8.8 IF2: 7.2 IF3: 7.2	IF1:65.6 IF2: 66.3 IF3: 65.9	1–4	Similar volume of formula and energy intake across IF groups and similar body weight.	[92]
5	Double-blind RCT	IF1:100:0 (*n =* 87) (whey without GMP) IF2: 60:40 (*n =* 87) BF: (*n =* 105)	IF1: 6.4 IF2: 8.6	IF1: 67.2 IF2: 64.6	3–12	IF1 = IF2 for protein and energy intake but IF2 >IF1 for weight (4–12 mo) and WAZ (12 mo). All IF >BF for WAZ from 5 mo.	[93]
6	Double-blind RCT	IF1:60:40 (*n =* 64) IF2: 70:30 (*n =* 72) α-LA enriched IF3: 70:30 (*n =* 68) GMP reduced BF: (*n =* 66)	IF1:8.8 IF2: 7.0 IF3:7.0	IF1:67.3 IF2: 68.2 IF3: 68.0	1–6	IF1 >IF2 and IF3 for energy and protein intake between 3 and 5 mo. IF groups similar for WAZ and weight gain (2–6 mo). IFs >BF for weight gain 2–6 mo. IFs >BF for WAZ from 4 to 6 mo.	[94]
7	Double-blind RCT	IF1: 30:70 (*n =* 50) IF2:70:30 (*n =* 51) BF: (*n =* 55)	IF1: 10.1 IF2: 7.2	IF1: 67 IF2: 67	0–4	IF1 >IF2 for volume and energy intake (1, 2, and 4 mo) and protein intake (1–4 mo), but similar weight gain.	[95]
8	Double-blind RCT	IF1:100:0 (*n =* 83) with partially hydrolyzed whey IF2: 60:40 (*n =* 80)	IF1:9.7 IF2: 8.5	IF1:66 IF2: 66	6–9	IF2 >IF1 for intake but similar body weight gain and WAZ.	[96]
9	Double-blind RCT	IF1:80:20 (*n =* 61) with partially hydrolyzed whey IF2: 60:40 (*n =* 67) BF: (*n =* 57)	IF1:8.0 IF2: 8.0	IF1:67.6 IF2: 67.6	0.5–6	IF2 >IF1 for volume and protein intake and IF1 >IF2 for weight gain and WAZ from 0.5 to 1 mo. IF1 >BF for WAZ from 1 to 6 mo.	[97]
10	Double-blind RCT	IF1:100:0 (*n =* 67) with partially hydrolyzed whey IF2: 60:40 (*n =* 76) BF: (*n =* 45)	IF1:8.1 IF2: 8.1	IF1:64 IF2: 64	0–6	IF1 = IF2 for intake and IF1 >IF2 for body weight gain but similar WAZ.	[98]
11	RCT	IF1:61:39 (*n =* 61) IF2: 63:37 (*n =* 65) with partially hydrolyzed whey BF: (*n =* 63)	IF1:8.6 IF2: 9.1	IF1:68 IF2: 68	0.5–3	IF1 = IF2 for intake, weight gain and WAZ at 1.6 and 3 mo. IF = BF for WAZ.	[99]
12	Double-blind RCT	IF1:100:0 (*n =* 158) with extensively hydrolyzed whey IF2: 20:80 (*n =* 157) BF: (*n =* 41)	IF1:7.2 IF2: 7.8	IF1:66 IF2: 66	0–6	IF1 >IF2 for feed intake only at 3 mo and body weight and WAZ were similar. IF groups >BF for WAZ at 1–3 mo.	[100]
13	Double-blind RCT	IF1:100:0 (*n =* 71) with extensively hydrolyzed whey IF2: 100:0 (*n =* 91) with partially hydrolyzed whey BF: (*n =* 83)	IF1:14 IF2: 11	IF1:67 IF2: 67	1–4	No differences in growth-related parameters between IF groups.	[101]
14	Open-label	IF=100:0 (*n =* 79) with hydrolyzed whey FO=100:0 (*n =* 79) with hydrolyzed whey BF: (*n =* 73)	IF: 7.6 FO: 6.4	IF:67 FO:67	0–12	IF/FO = BF for weight gain and WAZ during 12 mo.	[102]
15	Double-blind RCT	IF1:100:0 (*n =* 61) Partially hydrolyzed whey with prebiotic IF2: 100:0 (*n =* 46) Partially hydrolyzed whey with prebiotic IF3: 100:0 (*n =* 48) Partially hydrolyzed whey with prebiotic	IF1:7.2 IF2: 8.0 IF3:9.0	IF1:66 IF2: 66 IF3: 66	0.5–4	No differences in growth-related parameters except baseline to 4 mo difference in WAZ, which decreased in IF2.	[103]
16	Double-blind RCT	IF1: AA IF2: no data Extensively hydrolyzed whey proteins	IF1:10.7 IF2: 10.2	IF1:67 IF2: 62.5	0–12	IF1 = IF2 for protein and energy intake and WAZ.	[104]

Abbreviations: AA, amino acids; α-LA, α-lactalbumin; BF, breastfed; CN, casein; FO, follow-on formula; GMP, glycomacropeptide; IF, infant formula; LF, lactoferrin; WP, whey protein; WAZ, z scores for body weight-for-age.

**TABLE 4:** Impact of bovine whey proteins-to-casein ratio on growth outcomes of mammalian species in their suckling stage of life

#	Model	Strain	Age (d)	Type of milk proteins compared	Dose or % energy (E) of proteins in total diet or as part of metabolizable (ME) or digestible energy (DE)	Outcomes	Reference
1	Pigs	Pietrain X (large white X Landrance)	2–28	Liquid diets with high protein and low bovine WP:CN (58:42) ratio vs. low protein and high WP:CN (68:32) ratio	26% vs. 18% E	Protein intake increased by 26%E diet from day 4 but body weight increased from day 16	[105]
2	Pigs	Note available	2–16	Liquid diets with varied WP:CN ratios: (20:80), (40:60), (80:20) and (100:0)	250 mL diet/d/kg	Growth-rate increased by low ratio (40:60). Least efficient in converting feed into weight gain was (100:0)	[106]
3	Pigs	York X Landrace	15–43	Solid diet with varying WP:MP at (40:0), (30:10), (20:20), (10:30) and (0:40)	Not available	Feed to weight gain most efficient by 10:30 ratio and least by 40:0	[107]
4	Pigs	York X Landrace X Duroc	3–21	Liquid formulation comparing 100:0 vs. 60:40 WP:CN ratio	10% ME	The 60:40 ratio more efficient at converting feed to body weight gain. Plasma ghrelin and GLP-1 increased by high ratio	[108]
5	Pigs	Large White X Danish Landrace X Duroc	Preterm to 66 h	Liquid formulation comparing 100:0 vs. 40:60 WP:CN ratio	22% E from proteins	Plasma levels of galactose decreased by high ratio	[109]
6	Rats	Wister	14–21	Liquid rat milk vs. bovine milk	On mean 35% E from proteins from rat milk vs. 16% E from proteins in bovine milk	Body weight decreased in bovine milk–fed female rats and visceral fat decreased in bovine milk–fed male rats	[110]
7	Rat	Sprague-Dawley	7–14	Intragastric administration of bovine αCN, BSA, αLa or hydrolyzed αLa	150 mg = 1/20th of daily E at age 14	Serum GLP2 decreased by BSA compared with αCN. Serum GLP2 increased by hydrolyzed αLa compared unhydrolyzed	[111]
8	Mice	C57/BL6	1–21	Lactating mothers fed WP (100:0) or CN (0:100)	20% E proteins in the maternal diet	Body weight decreased in male and female suckling offsprings of lactating mothers fed the high WP ratio	[20]
9	Pigs	Large White X Danish Landrace X Duroc	1–5	Liquid bovine colostrum vs. sow’s milk	24–40 mL/kg/d	Intestinal villi height decreased by bovine colostrum	[112]
10	Pigs	Pietrain X (Finish Yorkshire X Belgian Landrance)	3–10	Sow milk replacer enriched with bovine WP vs. sow milk	Not available	Intestinal lactates and dipeptidylpeptidase activity decreased by bovine WP Intestinal length increased and villi height decreased by bovine WP	[113]
11	Pigs	Not available	0–28	Liquid bovine WPs vs. sow’s milk	Not available	Villi height decreased by bovine WP	[114]
12	Rats	Not available	5–15	Liquid bovine WP:CN ratios of 80:20, 60:40, 40:60 and 20:80 vs. mother-reared	Not available	Stomach, intestinal and hind gut weight decreased by high WP ratios	[115]
13	Rat	Sprague−Dawley	7–14	Human, caprine, and bovine milk proteins	90 mg/mL	Intestinal amino acid/peptide absorption least from bovine milk proteins	[116]

Abbreviations: α-LA, α-lactalbumin; BSA, bovine serum albumin; CN, casein; GLP, glucagon-like peptide; MP, milk proteins; WP, whey protein.

### Infant studies

In a study conducted by Fleddermann et al. [89], it was reported that infants offered IFs with a bovine WP-to-CN ratio of 60:40, delivering a protein-derived energy content of 8.8%, had a higher energy and protein intake but reduced weight gain compared with intake of same WP-to-CN ratio but with a lower protein content (7.6% by energy; #1 in Table 3). An intake that is not supporting a gain in weight could arise due to reduced nutrient absorption through the gut of infants offered bovine milk proteins and in ratios equivalent to human milk composition (WP:CN of 60:40). Infants offered bovine proteins with even higher (71:29) WP-to-CAS ratios had higher volume of intake compared with infants offered 62:38 ratio [90]. In this instance, the protein and energy intake were similar between the 2 groups as was the growth trajectory (WAZ) (#2, Table 3) [90]. Although the 71:29 ratio-group received marginally more (8%) protein-derived energy than the 62:38 ratio-group, receiving 7.7% of protein-derived energy, the total energy content in the higher (71:29) ratio-formula was lower (60 kcal/100 mL) than in the 62:38 ratio-formula, which provided 66 kcal/100 mL energy [90]. Thus, the higher volume of intake in the 71:29 ratio-group could arise due to the lower overall energy density in this formula, allowing infants to consume protein and energy intake and consequently, achieve a growth trajectory similar to that of 62:38 ratio-group receiving the more energy dense formula [90]. These data highlight the importance of macronutrient energy density in IF for growth outcomes of infants and how compensatory mechanisms act to increase intake to get sufficient nutrients to support growth when there is a reduced nutrient/energy availability in the feed. Extending the above work, Putet et al. [91] compared the growth-related effects of 10.8% with 7.2% protein-derived energy-IF, which had the same overall energy density (68 kcal/100 mL) as well as bovine WP-to-CN ratio (70:30). The study showed that infants provided with 10.8% protein-derived energy-IF consume more proteins (by 0.5 mo) and energy (by 2 mo), whereas weight gain and WAZ show delayed increases at month 4 (body weight) and month 6 (WAZ) compared with those offered the 7.2% energy protein-feed (#3, Table 3). There was no difference in body composition between the IF-fed groups. The studies by Fleddermann et al. [89] and Putet et al. [91] suggest that providing infants with formula with high quantities (>8% by energy) of bovine WP and CN in ratios equivalent to or exceeding 60:40 leads to an intake of proteins and energy that do not support weight gain and growth. On the basis of rodent and porcine studies detailed below, we propose that the discordance between intake and growth occurs because of a shift (reduction) in nutrient absorptive capacity of the gut after long-term intake (i.e., weeks to months) of high content of bovine WPs. In contrast, an acute intake, lasting several hours, allows the higher quantity of essential amino acids in bovine WPs to be digested and absorbed through the gut more readily than CN digestion [75,76]. Thus, in studies conducted by Fleddermann et al. [89] and Putet et al. [91] (#1 and #3 in Table 3), the infants offered high bovine WPs-enriched formula for 4–6 mo may have responded to the presumed reduced nutrient absorption by increasing protein intake and as a way to counteract the reduced [89] or delayed weight gain [91]. One may then assume that changing the protein combination (ratio and quantity) in IF should also change the growth outcomes. In fact, and in contrast to studies by Fleddermann et al. [89] and Putet et al. [91], which used fixed high ratios of bovine WP-to-CN and compared the effect on growth of increasing the protein content in formula, the study by Raiha et al. [92] investigated how the growth outcomes are affected in infants given a high bovine WP-to-CN ratio (70:30) with a low protein content (7.2% by energy) compared with those given a low ratio (60:40), delivering a high protein content (8.8% by energy) (#4, Table 3). In the latter study, there were no differences in intake and growth-related parameters between the 2 groups. The data support our suggestion that a WP-to-CN ratio of 60:40 or above and with a protein-derived energy of 8% or more is needed to cause the discordance between intake and growth compared with those offered the lower ratios/protein quantity.

Some insights into the mechanisms causing the discordance between intake and growth can be obtained from studies undertaken to investigate the effects of modified whey fractions on growth-related parameters. In these studies, the whey fractions were created with depleted glycomacropeptide (GMP) or enriched with α-lactalbumin (α-LA) (#5 and #6, Table 3) [93,94]. In a study by Tinghall Nilsson et al. [94], the growth-related effects of IF with a high bovine WP-to-CN ratio (70:30) and with depleted GMP, providing 7% energy from proteins, were compared with formula offered with unmodified bovine WP in low ratio (60:40), delivering 8.8% energy from proteins (#6, Table 3). The experimental setup was similar to aforementioned study by Raiha et al. [92] study, which used unmodified WPs and compared effects of a high ratio with low protein quantity with low ratio with a high protein quantity. Although Raiha et al. [92] found no differences in growth-related parameters between groups, the study by Tinghall Nilsson et al. [94] showed that infants offered IF with GMP-depleted whey fraction and in high (70:30) ratio with a low protein quantity (7% by energy) consumed less proteins and energy but were able to achieve a similar weight gain and WAZ scores to those offered unmodified whey fraction in low (60:40) ratio and high protein quantity (8.8% by energy) (#5 Table 3). We interpret these data to suggest that GMP in the whey fraction contributes to the discordance between intake and growth and that in the reduced availability of this specific protein in the formula, infants are able to absorb more nutrients and achieve a growth trajectory with less protein and energy consumed in the formula (#6 Table 3). In the study by Tinghall Nilsson et al. [94], the effects of whey fraction enriched with α-LA was also compared in a similar way. As for the GMP-depleted fraction, infants offered α-LA–enriched whey fraction in high ratio with low protein quantity consumed less proteins and energy and yet achieved a similar weight gain and WAZ scores to those offered unmodified whey fraction in low ratio and high protein quantity (#6, Table 3) [94]. We interpret these results to suggest that specific proteins in whey are responsible for reducing nutrient absorption through the gut (GMP), whereas others (α-LA) increase absorption and may cause minimal intake-growth discordance. Further studies are nonetheless needed to confirm this theory, particularly in light of the finding that a complete reversal of the ratio (70:30 compared with 30:70) did not change intake and growth in the expected way that would indicate a reduced nutrient absorption caused by bovine WP-enriched formula intake (#7, Table 3) [95].

Formulas containing partially hydrolyzed bovine WPs are given to infants who are unable to tolerate intact protein formulas, as the hydrolyzed proteins help reduce protein allergenicity [24,79]. The hydrolysis of WPs could also impact on mechanisms related to nutrient absorption, and exploring the effects of hydrolyzed compared with intact WPs could provide further insights into the related mechanisms that appear to cause the discordance between intake and growth mentioned in above studies. Indeed, consumption of IF enriched with hydrolyzed bovine WP was found to reduce intake compared with the group receiving unhydrolyzed proteins, whereas weight gain and WAZ scores were similar in the 2 groups (#8, Table 3) [96]. In another study, infants offered hydrolyzed WP formula consumed less and had reduced protein intake compared with infants receiving unhydrolyzed WP formula and yet the former group transiently (within the first month) gained more body weight (#9, Table 3) [97]. Otten et al. [98] showed that infants offered hydrolyzed WP-enriched formula gained more body weight despite similar intake to those receiving unhydrolyzed formula (#10, Table 3). These data suggest that prehydrolysis of WPs can, at least partially, correct the discordance between intake and growth seen with consumption of unhydrolyzed WPs, presumable because of greater absorption of amino acids (in the protein hydrolysate) through the proximal intestine, which can then be used for anabolic activities supporting body weight gain. In contrast, consumption of unhydrolyzed WPs-enriched formula over long term (i.e., weeks to months and as compared with intake of less amounts of the same proteins) may lead to some change in the digestive process to cause the potential reduced nutrient absorption, which we believe underlie the discordance between intake and growth-related parameters. In this regard, it is notable that unhydrolyzed bovine WPs cause compositional and functional pathway changes in the gut microbiota as compared with hydrolyzed WPs [117] and that long-term intake (5 compared with 17 wk) of unhydrolyzed bovine WPs in mice changed the composition of gut microbiota, alongside a reduction in ileal expression of neutral amino acid transporter, SLC6a19 over time (5 compared with 17 wk) and as compared with controls fed CN [21]. Perhaps, and as part of gut microbiota response to unhydrolyzed WPs intake, the protein-sensitive microorganisms may alter their digestive enzyme activities [118] to hydrolyze dietary WPs and use the peptides and amino acids to produce metabolites, which can then affect the nutrient absorptive capacity of the gut (e.g., nutrient transporter expression) [[19], [20], [21]]. There are contradictory results that dispute the effect of hydrolyzed bovine WP to potentially allow more nutrients to be used for growth [99,100], although the corresponding effects appear to be transient, seen only at a single time point (3 mo) in a study that was undertaken across a 6-mo period (#12, Table 3). Investigations that focused on the extent of hydrolysis (extensive compared with partial) did not reveal differences in efficacy (#13–#15, Table 3) [[101], [102], [103]]. There were also no differences in growth-related parameters between infants fed hydrolyzed formula and those supplemented with amino acids equivalent to protein quantity in the formula (#16, Table 3) [104]. The latter data suggest that the concentration of specific amino acids in the protein hydrolysate may be important in supporting weight gain. Conversely, in the unhydrolyzed WP form, the intestinal absorption of the same amino acids may be impeded by the dietary protein-induced changes in the gut microbiota, with capacity to utilize dietary amino acids, compared with hydrolyzed protein intake [117]. Together, these findings imply that infants given WP-enriched formula (60:40 or higher) in high protein quantities (≥8% by energy) have reduced nutrient absorption, and despite an increased intake to counter the presumed nutrients loss through excretion, gain less weight or gain a similar weight to those consuming a lower quantity of protein of the same ratio. The theory that infants attempt to correct nutrient loss from the body by increasing feed intake is consistent with the protein leverage hypothesis, which proposes that organisms must prioritize their protein intake and will consume more to gain proteins to sustain growth [119]. Thus, we suspect that infants fed a high bovine WP-enriched formula over a period of time (i.e., weeks to months) may have reduced amino acid absorption, and possibly also other nutrients including fatty acids and carbohydrates in light of the finding that mice fed bovine WPs show greater triacylglycerol content in feces and increased caecal availability of carbohydrate-breakdown products (e.g., pyruvate) compared with controls fed CN [19,20]. Prehydrolysis of bovine WPs appears to improve growth outcomes presumably by allowing more amino acids to be absorbed by the proximal part of the intestine, thereby limiting protein constituent (i.e., amino acids and peptides)-access to gut microbiota, more abundant in the distal gut [120] and in turn, limit the microbial activity to produce metabolites, which can affect the gut capacity to absorb nutrients [[19], [20], [21]].

### Animal studies

Piglets fed a diet with 26% energy from proteins and with a 58:42 bovine WP-to-CN ratio were shown to increase protein intake by day 4 and weight gain (much later) by day 16 compared with controls receiving a lower (18%) energy protein-diet with a 68:32 bovine WP-to-CN ratio (#1: Table 4) [105]. One interpretation of the data is that the higher intake in piglets consuming the low bovine WP-to-CN ratio facilitates assimilation of amino acids, many of which are more abundant in bovine WP than CN [75], and use these amino acids to sustain growth. Another interpretation of the data is that the higher quantity of bovine WP consumed in the low WP-to-CN ratio-diet was sufficient to reduce nutrient absorption in piglets and consequently, they compensate by increasing intake (by day 4) to sustain growth, leading to delayed increase in body weight (by day 16) compared with controls receiving a lower quantity of proteins with a higher WP-to-CN ratio. The latter interpretation is opposite to that proposed for infants, because humans in the infancy stage appear to respond to high WP-to-CN ratio and in high protein quantities, leading to the discordance between intake and growth. The discrepancy between pig and human-responses may be due to species differences in WP and CN delivered through mother’s milk (e.g., 50:50 in late lactation human milk, whereas 47:53 in late lactation in sow’s milk; Table 1) and digestive enzyme activities existing in the 2 species (e.g., humans do not have the gene for chymosin, whereas pigs do; Table 2), and thus, any subtle increase in the ratio between WP and CN in the feed could generate a greater response in the porcine gut compared with human gut, particular when given in high protein quantities. The latter explanation is supported by other studies testing different ratios of bovine WP-to-CN ratio and, in each case, ratios with a high bovine WP content were least efficient in converting feed into body weight gain (#2–#4, Table 4) [[106], [107], [108]]. The impact of bovine WP on nutrient absorption is reflected by reduced galactose availability in plasma after oral administration of a bolus (15 mL/Kg) of 5% galactose and 2% mannitol solution (#5, Table 4) [109] and increased plasma availability of 2 hormones known to have opposing effects on energy balance, with one known to cause a nutrient surplus (ghrelin), whereas the other generating a nutrient deficit [glucagon-like peptide (GLP-1)] [108,121,122]. Whether the effects of one hormone are greater than the other to induce the potential reduced nutrient absorption remains to be determined.

Studies undertaken in rats and mice add further support to the theory that bovine WPs are the cause of reduced nutrient absorption. A comparison of effects of bovine and rat milk on growth outcomes of rat pups shows that the former causes sex-dependent alteration in body weight and composition [110]. Notably, female rat pups fed bovine milk reduced body weight gain compared with controls fed rat milk, whereas male rat pups fed bovine milk show no significant changes in weight gain but had reduced retroperitoneal fat compared with controls fed rat milk (#6, Table 4). The effects on body weight and adiposity were seen in pups receiving the same volume of milk (20 mL/kg/d) but with different milk macronutrient compositions and energy densities of the milk, reflecting the changes in milk during mid-to-late lactation in the 2 species [110]. To isolate the effects of bovine WPs from potential confounding changes in other macronutrients, we recently fed mice bovine WPs or CN as the sole protein source in diets matching all other macronutrients [20]. The study used lactating murine mothers and their pups. Feeding bovine WPs to lactating mothers reduced their body weight gain compared with mothers fed CN. The bovine WP-effects in the maternal diet also reduced body weight gain of the suckling pups compared with pups of mothers fed CN (#8, Table 4) [20]. Because the lactating murine mothers fed bovine WP had similar food (and energy) intake and increased caecal abundance of carbohydrate-breakdown products (e.g., pyruvic acid and lactic acid) and yet reduced weight gain compared with CN-fed controls, the data suggest that the bovine WPs cause reduced nutrient absorption in the parental generation, and that this effect might also be passed onto the offspring to cause their reduced weight gain during the suckling stage. In support of the latter suggestion, male pups of mothers fed bovine WPs had increased caecal abundance of glycine, valine, leucine, threonine, serine, tyrosine, cysteine, and phenylalanine compared with male pups raised by mothers fed CN [20]. The reduced weight gain in the suckling young extended into postweaning stage (#8, Table 4). In the postweaned female offspring that had a history of maternal WP intake, the subcutaneous adipose tissue decreased in weight compared with female offspring of mothers that had consumed CN [20].

Extended work on mice and pigs show that high WP-to-CN ratios reduce gut size and villi height and lactase and amino and dipeptidase activities in the intestine (#9–#12, Table 4) [[112], [113], [114], [115]], as well as decreased amino acid and peptide absorption through the intestine compared with lower ratios (#13, Table 4) [116]. These effects may be related to the bovine serum albumin (BSA) in the whey fraction, as rat studies show that this protein (compared with CN) reduces plasma levels of GLP-2, which has roles in intestinal cell proliferation and nutrient absorption (#7, Table 4) [111,123]. In contrast, prehydrolyzed α-LA compared with intact protein was found to increase plasma levels of GLP-2 in suckling rats [111], raising the possibility that the hydrolysis of bovine WPs improves nutrient absorption, at least in part, because of the bioactivity in α-LA released by protein hydrolysis, which can impact on GLP-2 production and, in turn, increase nutrient absorptive capacity of the gut [111,123].

## Understanding the Mechanisms

The long-term intake (i.e., weeks to several months) of bovine WPs during adulthood is known to cause a reduction in body weight and adiposity in humans and other mammalian species compared with intake of many other dietary proteins such as soy proteins [120,124]. On the basis of rodent studies, and compared with bovine CN, the WP effect, which reduces body weight gain, appears to result from reduction in nutrient absorption, affecting the absorption of amino acids, fatty acids, and carbohydrates [[19], [20], [21]]. This contrasts with acute bovine WP challenges, lasting for several hours, which shows that bovine WPs are digested and the associated amino acids absorbed more readily than bovine CN [75,76]. The difference in acute compared with chronic effects of intake of bovine WPs could be due to the protein duration-dependent changes in the composition of gut microbiota [21]. In this regard, it is notable that in mice, 5 wk of bovine WP intake led to reduced plasma levels of methionine compared with controls fed bovine CN, whereas extending the feed intake to 17 wk caused WP group to reduce plasma levels of methionine, threonine, and tyrosine alongside a reduction in ileal expression of neutral amino acid transporter, SLC6a19 compared with controls fed CN [21]. A difference in plasma levels of amino acids could arise due to an altered protein digestion, caused by differences in dietary protein-induced changes in production or activity of gut microbial–derived digestive enzymes [118]. There are murine data to support the latter suggestion [19]. In the latter study, we showed that intake of bovine WPs for 12 wk increased gut microbiota pathway-activities related to dietary protein/amino acid degradation compared with CN intake [19]. The microbial changes were mirrored in the amino acid abundance in the ceacal contents [19]. Fecal transplant experiments confirmed the efficacy of gut microbial pathways related to dietary protein/amino acid degradation to reduce weight gain in recipient mice, suggesting that the effects of bovine WPs on gut size and nutrient absorption are mediated, at least in part, by (certain components of) the gut microbiota [19]. This work prompted us to assess if the same proteins impact on growth outcomes of suckling murine pups. As detailed above, the experimental design involved feeding bovine WP or CN to lactating mice that we know to have a reduced WP content than CN in milk (39:61) (Table 1). We reasoned that feeding bovine WP to lactating mice would reduce nutrient absorption in the mothers compared with controls fed CN and that this effect would be transferred into the suckling pups to affect their growth. Indeed, evidence from rodent and human studies suggests that the protein quality and quantity in maternal diet [i.e., wheat compared with CN and high egg intake (>4 eggs/wk) compared with egg-free diet] affect milk composition including the ratio between endogenous WP and CN [35,125], which is expected to impact on growth outcomes of the suckling young. In addition to this mechanism, a role for microbiota in mediating health outcomes of milk should not be ignored in light of the findings that vertical transmission of bifidobacterial species was tracked from parent’s milk onto infants [126] and that activities of bifidobacterial species in tri-association (i.e., *B. bifidum* PRL2010, *B. breve* PRL2012, and *B. longum* PRL2022) were found to increase gene expression associated with epithelial cell integrity in human CaCo-2/HT-29-MTX cells compared with coculturing of individual species [127]. In our murine study, lactating mothers fed bovine WP were found to have reduced gut weight compared with controls fed CN and this effect was engrafted onto offspring that had unchanged food intake but reduced weight gain [20]. Metabolomic analysis of caecal contents revealed evidence of increased caecal availability of amino acids (glycine, valine, leucine, threonine, serine, tyrosine, cysteine, and phenylalanine) in male pups of mothers fed bovine WP compared with male pups raised by mothers fed CN [20]. Although the latter study did not investigate the composition of the gut microbiota, based on our previous data from adult mice [19], we propose that an increased availability of dietary amino acids in the ceacal contents in the offspring of mothers fed bovine WP is a representation of microbial activity to degrade and utilize dietary proteins and that these effects would have been passed on from the previous generation. Whether any vertical transmission of microbial activity is due to a change in protein or microbiota composition in milk or both, with each affecting the gut microbiota in the murine offsprings remains to be determined. It is also notable that gut microbiota composition differs between human milk and formula-fed infants, with former having a high abundance of bifidobacteria (in particular *Bifidobacterium breve*, *Bifidobacterium bifidum*, and *Bifidobacterium longum*) and *Lactobacillus* species compared with formula-fed infants, whereas IF-feeding increasing the abundance of *Staphylococcus* and *Clostridium* compared with human milk intake [58,[62], [63], [64],^128^]. These results suggest that the differences in protein composition between breast milk and IF can cause distinct effects on the composition of the gut microbiota, and together with data from a rat study showing that varying bovine WP-to-CN ratios affect composition of the gut microbiota [129], we propose that the microbial effects (i.e., production of metabolites) affect the metabolic activity of gut cells in infants, causing a reduction in nutrient absorptive capacity and thereby lose nutrients through feces. Specific proteins within the bovine whey fraction, such as BSA and GMP, may be responsible for the reduced nutrient absorption, whereas others may have an opposite effect (e.g., α-LA). Their hydrolysis appears to affect nutrient absorption (Table 4). We suspect that this may be due to greater absorption of amino acids by the small intestine after intake of hydrolyzed bovine WP, thus reducing the amino acid availability to the gut microbiota to produce metabolites, which can cause the reduction in absorptive capacity of the gut.

## Physiological Perspective and Future Directions

Mammals must regulate their energy intake and expenditure to maintain bodily functions and to sustain growth. The energy-balance–related mechanisms include the gut microbiota and gut-associated nutrient transporters and sensing mechanisms including those that produce GLP-1 and -2 [123,130]. Although much of the focus to understand how these components act relied on studies undertaken in the adolescence and/or adult life stages, we propose that infancy provides another opportunity to explore how the energy balance is regulated, particularly in developing tissues and in response to a specific nutrient input (i.e., parent’s milk). We propose that the host and gut microbial–derived digestive enzymes allow endogenous milk proteins, found in different ratios in each mammalian species, to be digested and absorbed to support growth of the host. Disruption or alteration of the interaction between diet (milk composition), microbiota and nutrient absorption by cross-transfer of milk proteins is expected to affect body weight gain. The mechanism may involve an alteration in the composition of the gut microbiota after consumption of nonspecies-specific milk proteins, as that shown to occur in infants fed IF as compared with breastmilk intake [58,[62], [63], [64],^128^] and rats fed varying ratios of bovine WP-to-CN [129], leading to altered protein digestion in the gut lumen and production of microbial-derived metabolites [19,120], which can affect nutrient absorptive capacity of the gut, similar to that occurring in mice fed bovine WPs compared with bovine CN [[19], [20], [21]]. Prehydrolysis of milk proteins mitigates the growth-related outcomes of high bovine WP-to-CN ratios, presumably because of greater intestinal absorption of amino acids, and reduced accessibility of these nutrients for gut microbiota involved in the production of metabolites, which can impact on the nutrient absorptive capacity of the gut. Use of α-LA–enriched formula also mitigates the presumed reduced nutrient absorption seen with intake of intact whey fraction, and based on data collected from rodent studies, the mechanism may involve an increased production of GLP-2, which increases intestinal cell proliferation and tissue-nutrient absorptive capacity [123,130]. Further studies are needed to verify the extent of bovine WP-induced reduction in nutrient absorption by measuring the energy content and composition in feces and by understanding how the protein composition of intact compared with hydrolyzed forms affects the composition of the gut microbiota and how the gut microbial activity (i.e., through production of metabolites) is then communicated to the gut to affect cellular activity. Beyond nutrient absorption, we also have little knowledge of how any absorbed nutrients are used and/or stored in different tissues including in the muscle and adipose. Future studies should also seek to determine if the effects detailed here are restricted to bovine WPs or if proteins sourced from other species have similar impact to dissociate intake and growth outcomes when fed to a different species.

On the basis of data presented here, we conclude that intake of formulas containing a bovine WP-to-CN ratio of 60:40 or higher, when consumed at ≥8% of total energy intake, results in a reduced nutrient absorption through the gut, leading to increased nutrient loss through feces, compromising overall growth efficiency. To improve nutrient absorption, incorporating hydrolyzed bovine WPs or selectively using specific WP fractions such as α-LA could be promising strategies. Further studies are required to determine how long-term intake of bovine WP-enriched formula affects the gut microbiota and how the resulting changes are communicated to the gut cells to affect nutrient absorption. These studies may enable the formulation of IFs that maintain a healthy growth trajectory and will likely utilize protein contents below the current regulatory standards of 1.8 g/100 kcal, thereby optimizing nutritional quality without excessive protein burden.

## Author contributions

The authors’ responsibilities were as follows – KNN: concept; KNN, DvS, PDC: conducted search; KNN, DvS, PDC: analyzed data; KNN, DvS, PDC: wrote paper; KNN: responsibility for final content; and all authors: have read and approved the final manuscript.

## Data availability

Data mentioned in this manuscript were derived from public domain resources.

## Declaration of Generative AI and AI-Assisted Technologies in the Writing Process

The authors declare that no generative AI or AI-assisted technologies were used in the writing of this manuscript.

## Funding

The work was supported by Research Ireland (formerly, Science Foundation Ireland) and the Department of Agriculture, Food and Marine, under the grant 16/RC/3835 (VistaMilk). Funding source has no restrictions regarding publication.

## Conflicts of interest

KNN received support from the American Dairy Product Institute to attend and contribute to conferences. PDC has a relationship with SeqBiome Ltd that includes equity or stocks. PDC has received research funding from FrieslandCampina, PrecisionBiotics Group, PepsiCo, and Danone. PDC has received support from PepsiCo, Abbott, Arla, Danone, Yakult, AG1, the National Dairy Council (United States), and H&H to attend and speak at scientific conferences and other events. DvS’s research work has received financial support from Danone/Nutricia, FrieslandCampina, DSM-Firmenich, and Nestle.
